# Mental distress and climate-related coastal hazards: Evidence from national studies in Indonesia

**DOI:** 10.1016/j.joclim.2026.100699

**Published:** 2026-05-22

**Authors:** Mashita Fajri, Reynalda Fildzah Dessyrianti, Sujarwoto Sujarwoto, Herni Susanti, Asri Maharani

**Affiliations:** a Department of Psychiatric Nursing, Faculty of Nursing, Universitas Indonesia, Depok 16424, Indonesia; b School of Psychology, Faculty of Psychology, Universitas Ciputra Surabaya, Surabaya 60219, Indonesia; c Portsmouth Brawijaya Center for Global Health, Population and Policy and Department of Public Administration, Universitas Brawijaya, Malang 65145, Indonesia; d Division of Nursing, Faculty of Biology, Medicine, and Health, University of Manchester, Manchester M13 9PL, United Kingdom

**Keywords:** Climate change, Mental health, Coastal hazards, Mental distress

## Abstract

**Introduction:**

Climate change has a significant impact on mental health and psychological well-being worldwide. However, the mental health consequences of rising seas linked to climate change remain limited, particularly in Indonesia. This paper aims to evaluate the risk of mental distress among individuals residing in coastal regions susceptible to climate-related natural hazards.

**Method:**

This study utilised data from 642,419 adults who participated in the 2018 Indonesia Basic Health Survey (Riskesdas). The correlation between residing in coastal hazard zones and mental distress was analysed using a multivariable logistic regression. The analyses were adjusted for sociodemographic factors, health status, and access to healthcare.

**Results:**

Approximately 201,109 (31.30%) live in coastal hazards area. Our analysis indicates that residents of coastal hazard areas have 1.16 times (95%CI: 1.01 – 1.33) increased likelihood of mental distress compared with non-coastal residents. Additionally, living in districts impacted by coastal abrasion and hurricane-prone areas was associated with slightly elevated odds of mental distress, 1.01 (95% CI: 1.00–1.03) and 1.00 (95% CI: 1.00–1.01), respectively. Factors associated with lower odds of mental distress include older age, higher education, being married, being employed or retired, higher household expenditure, and healthy lifestyle behaviours. In contrast, higher odds were observed among females, divorced individuals, active smokers, those with comorbidities, and individuals facing barriers to healthcare or family mental illness.

**Conclusion:**

Residing in coastal hazard areas correlates with higher odds of mental distress. Targeted mental health interventions are essential to support these at-risk groups and lessen climate-related health risks.

## 1 Introduction

Climate change poses major global challenges with profound environmental and health impacts [1]. Rising temperatures are causing rising sea levels through melting ice and warming oceans, threatening coastal communities globally [2,3]. Indonesia, the largest archipelagic nation, has approximately 150 million inhabitants along its coastlines, which are highly vulnerable to erosion, tidal flooding, and tropical storms [[4], [5], [6]]. The loss of land, infrastructure damage, and home displacement have serious economic and emotional consequences [4,6]. Beyond physical and material losses, the ongoing threats of environmental hazards fuel chronic stress, anxiety, and uncertainty [7,8]. Understanding the impact of environmental change on mental health is crucial for developing support and adaptation strategies for vulnerable coastal communities.

An increasing amount of research has highlighted the psychological impacts of climate change [[9], [10], [11], [12]], Particularly through the rising occurrence and intensity of extreme weather events, such as floods, heatwaves, and wildfires [9]. These climate-related stressors have been consistently associated with elevated psychological distress [9,13]. Evidence further suggests that people with pre-existing mental health conditions are especially vulnerable, facing higher risks of morbidity and mortality during climate-related events [14,15].

While the health impacts of climate change are increasingly recognised, evidence on its mental health consequences remains limited in many high-risk settings, especially in lower- and middle-income levels (LMICs) [10,16]. In Indonesia, the psychological effects of climate-related hazards are understudied. Existing studies mostly focus on acute disasters or small-scale qualitative cases that do not reflect population-level trends [[17], [18], [19]]. To date, only one national study has examined the link between coastal hazards and mental health, focusing only on depression [8]. Major gaps persist: no studies have used validated tools such as the Self-Reporting Questionnaire (SRQ)-20 to assess mental distress; few have examined coastal hazards’ effects on distress; and none have applied multilevel modelling to explore individual vulnerabilities and district-level exposures.

This study addresses these gaps by investigating the relationship between coastal hazards and mental distress among Indonesian adults. We used nationally representative datasets, the 2018 Basic Health Survey (Riskesdas) and 2018 Village Potential Statistics (PODES), to assess whether coastal residents face greater mental distress than non-coastal populations. This study provides a foundation for integrating mental health into climate adaptation and public health strategies for Indonesia’s coastal communities, thereby addressing mental health needs that are crucial for effective intervention.

## 2 Methods

### 2.1 Study design

This study used a cross-sectional design using Riskesdas and Podes 2018 datasets. Riskesdas covers all 34 provinces and 514 districts using multistage systematic random sampling [20]. For this analysis, we included adults with complete responses to depression-related items, yielding a final sample of 642,419 individuals.

Data from the Riskesdas 2018 were then linked with data from the Podes 2018. Podes collects information at the village level in Indonesia [21]. While Podes village-level information, this study utilised district-level (kabupaten/kota) aggregates. Podes 2018 data were used to determine whether coastal districts experienced hazards (abrasion, hurricanes, and tidal flooding) between 2015 and 2017, based on village reports aggregated using Indonesian Statistical identifiers. These district-level hazard indicators were merged with individual-level Riskesdas 2018 data using standardised district codes, creating a nested dataset of individuals within districts. Hazard variables were therefore treated as district-level contextual exposures. Although this approach is common in studies linking Riskesdas and Podes data [8], it may not fully capture within-district variation.

### 2.2 Ethical clearance

Ethical approval for Riskesdas 2018 was obtained from the Ethics Committee of the NIHRD, Ministry of Health, Indonesia (No. LB.02.01/2/KE.267/2017), with written informed consent obtained from all participants. PODES 2018, collected by Statistics Indonesia (BPS) under Law No. 16/1997, is publicly available in anonymised. No additional ethical approval was required for this secondary data analysis.

### 2.3 Measures of mental distress

Mental distress was assessed using the SRQ-20, a standardised screening tool for common mental disorders in community and primary care [22]. The SRQ-20 comprises 20 yes/no questions to capture anxiety, depressive, and somatic symptoms experienced in the past month (e.g., persistent sadness, sleep disturbance, fatigue, difficulty concentrating). The instrument demonstrated high internal consistency in this study (Cronbach’s α = 0.86). Following USAID guidelines, a cut-off score of ≥10 was used to indicate mental distress. A sensitivity analysis using a lower cutoff of ≥6, as validated for the Indonesian population by Idaiani et al. [22], was conducted to evaluate robustness (Supplementary Table S1).

### 2.4 Measures of coastal hazards

Districts were classified as coastal if they included villages directly along the coast. Coastal abrasion refers to shoreline retreat due to wave-driven sediment movement [23]; tidal flooding to inundation of low-lying coastal areas during high tides, often intensified by sea-level rise [24]; and hurricanes to intense tropical cyclones with sustained winds ≥64 knots (118 km/h) [25]. Respondents were classified as living in a coastal hazard–affected district if at least one hazard occurred during the study period.

### 2.5 Covariates

Covariates were grouped into five domains: demographic (age, sex), socio-economic (education, marital status, employment, household expenditure quintiles), health behaviour (smoking, alcohol consumption, physical activity), health status (self-reported chronic conditions), and household characteristics (having a family member with psychosis). Access to healthcare was measured using a principal component analysis (PCA) index based on transportation mode, travel time, and cost, dichotomised into “easy” versus “difficult” access. All covariates were derived from standard Riskesdas items and reflect established determinants of mental health in prior population-based studies using the same dataset [8,26].

### 2.6 Statistical analysis

Respondents' characteristics were summarised using frequencies and percentages. Bivariate associations between mental distress and sociodemographic characteristics among residents of hazard-affected districts were examined using χ² tests (chi-square analyses).

Multilevel logistic regression was used to calculate odds ratios (ORs) and 95% confidence intervals (CIs) for mental distress, with a random intercept at the district level. Models examined associations between mental distress and exposure to coastal hazards, adjusting for covariates described above. Hazard-specific models were estimated separately to avoid collinearity, as hazards often co-occur geographically; these analyses do not distinguish between single- and multi-hazard exposure. The analyses used adaptive Gauss–Hermite quadrature (five points) and were conducted in Stata 18.0.

## 3 Results

### 3.1 Characteristics of respondents

The analysis included 642,452 adults across Indonesia, of whom 201,109 (31.3%) lived in districts exposed to coastal hazards (Table 1). The prevalence of mental distress was significantly higher in hazard-prone areas (3.25%) than in non-hazard areas (2.56%) (p < 0.001). Descriptive analysis showed significant associations between distress and covariates. Mental distress was more common among middle-aged adults, particularly those aged 45–54. Women accounted for 63.96% of distressed individuals in coastal areas. Educational attainment was inversely related to distress, with 64.48% of cases reporting only primary education or less.

**Table 1:** Characteristics of all respondents categorized by residential area (with or without coastal hazards).

Variables	All respondents			Respondents living in coastal hazard districts (N= 201,109)		
	Non-coastal hazard districts (N=441,343)	Coastal hazard districts (N=201,109)	p-value*	Not mentally distressed (N = 192,604)	Mental distressed (N = 6,534)	p-value*
Mental distress			<0.001			<0.001
No	425,855 (96.49%)	192,604 (95.77%)				
Yes	11,292 (2.56%)	6,534 (3.25%)				
Age			<0.001			<0.001
18-24 years old				25,971 (13.48%)	837 (12.81%)	
25–34 years old	59,746 (13.54%)	26,962 (13.41%)		40,680 (21.12%)	1,051 (16.09%)	
35–44 years old	90,473 (20.50%)	41,953 (20.86%)		45,748 (23.75%)	1,432 (21.92%)	
45–54 years old	104,336 (23.64%)	47,358 (23.55%)		38,035 (19.75%)	1,377 (21.07%)	
55–64 years old	58,999 (13.37%)	39,594 (19.69%)		24,991 (12.98%)	948 (14.51%)	
65–74 years old	26,677 (6.04%)	12,909 (6.42%)		11,994 (6.23%)	551 (8.43%)	
75 years old or above	12,736 (2.89%)	6,145 (3.06%)		5,185 (2.69%)	338 (5.17%)	
Sex			<0.001			<0.001
Female	231,287 (52.41%)	106,431 (52.92%)		101,163 (52.52%)	4,179 (63.96%)	
Male	210,023 (47.59%)	94,678 (47.08%)		91,441 (47.48%)	2,355 (36.04%)	
Education			<0.001			<0.001
Primary or lower	191,904 (43.48%)	98,072 (48.77%)		92,242 (47.89%)	1,367 (64.48%)	
Junior high school	79,851 (43.48%)	32,617 (16.22%)		31,533 (16.37%)	954 (14.60%)	
Senior high school	169,555 (38.42%)	70,420 (35.02%)		68,829 (35.74%)	4,213 (20.92%)	
Marital status			<0.001			<0.001
Single	68,421 (15.50%)	30.423 (15.13%)		28,894 (15.00%)	940 (14.39%)	
Married	329,056 (74.56%)	151,013 (75.09%)		145,928 (75.77%)	4,449 (68.09%)	
Divorced and a widower	43,833 (9.93%)	19,673 (9.78%)		17,782 (9.23%)	1,145 (17.52%)	
Occupation			<0.001			<0.001
Employed or retired	56,378 (12.77%)	21,946 (10.91%)		21,633 (11.23%)	287 (4.39%)	
Self-employed	66,687 (15.11%)	24,635 (12.25%)		24,035 (12.48%)	547 (8.37%)	
Informal worker	149,199 (33.81%)	75,227 (37.41%)		72,380 (37.58%)	2,572 (39.36%)	
Others	27,415 (6.21%)	15,663 (7.79%)		15,175 (7.88%)	443 (6.78%)	
Jobless	128,695 (29.16%)	57,798 (28.74%)		53,742 (27.90%)	2,515 (38.49%)	
Student	12,936 (2.93%)	5,840 (2.90%)		5,639 (2.93%)	170 (2.60%)	
Quintile			<0.001			<0.001
1^st^ Quintile	72,460 (16.42%)	41,935 (20.85%)		39,539 (20.53%)	1,785 (27.32%)	
2^nd^ Quintile	80,993 (18.35%)	40,228 (20.00%)		38,301 (19.89%)	1,502 (22.99%)	
3^rd^ Quintile	87,415 (19.81%)	39,936 (19.86%)		38,265 (19.87%)	1,293 (19.79%)	
4^th^ Quintile	94,449 (21.40%)	40,326 (20.05%)		38,881 (20.19%)	1,143 (17.49%)	
5^th^ Quintile	105,993 (24.02%)	38,684 (19.24%)		37,618 (19.53%)	811 (12.41%)	
Smoking status			<0.001			<0.001
Everyday	118,580 (26.87%)	54,712 (27.21%)		53,012 (27.52%)	1,428 (21.85%)	
Not everyday	20,187 (4.57%)	9,706 (4.83%)		9,277 (4.82%)	356 (5.45%)	
Ex-smoker	24,107 (5.46%)	9,630 (4.79%)		9,066 (4.71%)	394 (6.03%)	
Not smoker	278,436 (63.09%)	127,061 (63.18%)		121,249 (95.43%)	4,356 (66.67%)	
Alcohol consumption			<0.001			<0.001
Under standard	11,145 (2.53%)	6,685 (3.32%)		6,387 (3.32%)	278 (4.25%)	
More than standard	6,261 (1.42%)	6,420 (3.19%)		6,105 (3.17%)	302 (4.62%)	
No alcohol	423,904 (96.05%)	188,004 (93.48%)		180,112 (93.51%)	5,954 (91.12%)	
Physically active			<0.001			<0.001
Less active	43,399	20,216		19,379 (10.06%)	717 (10.97%)	
Active	397,911	180,893		173,225 (89.94%)	5,817 (89.03%)	
Have comorbidities			<0.001			<0.001
No comorbid (0)	349,380 (79.16%)	161,445 (80.28%)		155,978 (80.98%)	4,167 (63.77%)	
Low comorbidities (1-3)	91,029 (20.63%)	39,284 (19.53%)		36,339 (18.87%)	2,301 (35.22%)	
High comorbidities (4-8)	901 (0.20%)	380 (0.19%)		287 (0.15%)	66 (1.01%)	
Have a family member with psychosis			<0.001			<0.001
No	437,596 (99.15%)	199,287 (99.09%)		191,197 (99.27%)	6,369 (97.47%)	
Yes	3,714 (0.84%)	1,822 (0.91%)		1,407 (0.73%)	165 (2.53%)	
Have difficulty accessing healthcare			<0.001			<0.001
No	361,986 (82.02%)	150,424 (74.80%)		144,946 (75.26%)	4,032 (61.71%)	
Yes	40,706 (9.22%)	24,654 (12.26%)		23,191 (12.04%)	1,230 (18.82%)	

Unmarried and unemployed respondents reported higher distress; 38.49% of distressed residents were unemployed. Those in the lowest expenditure quintile had the highest prevalence (27.32%), with rates declining across higher quintiles. Non-smokers and ex-smokers reported more distress than daily smokers, and heavy drinkers had slightly higher distress than moderate drinkers. Physical inactivity, chronic illness (especially multimorbidity), and family history of psychosis were also linked to elevated distress. Finally, those reporting difficulty accessing healthcare showed a strikingly higher prevalence (18.82%).

### 3.2 Multilevel logistic regression analysis

As shown in Table 2, living in a coastal hazard-affected district was associated with significantly higher odds of experiencing mental distress (OR: 1.16; 95% CI: 1.01–1.33; p<0.05), after adjusting for covariates. Coastal abrasion and hurricanes are modestly associated with mental distress (OR: 1.01; 95% CI: 1.00–1.03; p<0.05 and (OR: 1.00; 95% CI: 1.00–1.01; p<0.05). In contrast, tidal flooding was not significantly correlated with mental distress.

**Table 2:** Results of multivariable logistic regression analysing the relationship between residing in coastal hazard areas and mental distress.

Variables	Model 1‡	Model 2‡	Model 3‡	Model 4‡
Living in coastal hazards	1.16 (1.01 – 1.33)*			
Living in district with coastal abrasion		1.01 (1.00 – 1.03)*		
Living in district area with hurricanes			1.00 (1.00 – 1.01)*	
Living in district with tidal flooding				1.00 (0.99 – 1.02)
Age group (Ref: 18-24 years old)				
25–34 years old	0.90 (0.84 – 0.97)*	0.90 (0.84 – 0.97)*	0.90 (0.84 – 0.97)*	0.90 (0.84 – 0.97)*
35–44 years old	0.94 (0.88 – 1.02)	0.94 (0.88 – 1.02)	0.94 (0.88 – 1.02)	0.94 (0.88 – 1.02)
45–54 years old	0.91 (0.85 – 0.99)*	0.91 (0.85 – 0.99)*	0.91 (0.85 – 0.99)*	0.91 (0.85 – 0.99)*
55–64 years old	0.72 (0.67 – 0.79)⁎⁎⁎	0.72 (0.67 – 0.79)⁎⁎⁎	0.72 (0.67 – 0.79)⁎⁎⁎	0.72 (0.67 – 0.79)⁎⁎⁎
65–74 years old	0.69 (0.63 – 0.76)⁎⁎⁎	0.69 (0.63 – 0.76)⁎⁎⁎	0.69 (0.63 – 0.76)⁎⁎⁎	0.69 (0.63 – 0.76)⁎⁎⁎
75 years old or above	0.78 (0.70 – 0.88)⁎⁎⁎	0.78 (0.70 – 0.88)⁎⁎⁎	0.78 (0.70 – 0.87)⁎⁎⁎	0.78 (0.70 – 0.88)⁎⁎⁎
Sex (Ref: male)				
Female	1.98 (1.87 – 2.10)⁎⁎⁎	1.98 (1.87 – 2.10)⁎⁎⁎	1.98 (1.87 – 2.10)⁎⁎⁎	1.98 (1.87 – 2.10)⁎⁎⁎
Education (Ref: Primary or lower)				
Junior high school	0.76 (0.72 – 0.79)⁎⁎⁎	0.76 (0.72 – 0.79)⁎⁎⁎	0.76 (0.72 – 0.79)⁎⁎⁎	0.76 (0.72 – 0.79)⁎⁎⁎
Senior high school	0.57 (0.54 – 0.59)⁎⁎⁎	0.57 (0.54 – 0.59)⁎⁎⁎	0.57 (0.54 – 0.59)⁎⁎⁎	0.57 (0.54 – 0.59)⁎⁎⁎
Marital status (Ref: Single)				
Married	0.67 (0.62 – 0.71)⁎⁎⁎	0.67 (0.62 – 0.71)⁎⁎⁎	0.67 (0.62 – 0.71)⁎⁎⁎	0.67 (0.62 – 0.71)⁎⁎⁎
Divorced or widowed	1.05 (0.97 – 1.14)	1.05 (0.97 – 1.14)	1.05 (0.97 – 1.14)	1.05 (0.97 – 1.14)
Occupation (Ref: Jobless)				
Employed or retired	0.60 (0.56 – 0.65)⁎⁎⁎	0.60 (0.56 – 0.65)⁎⁎⁎	0.60 (0.56 – 0.65)⁎⁎⁎	0.60 (0.56 – 0.65)⁎⁎⁎
Self-employed	0.80 (0.75 – 0.85)⁎⁎⁎	0.80 (0.75 – 0.85)⁎⁎⁎	0.80 (0.75 – 0.85)⁎⁎⁎	0.80 (0.75 – 0.85)⁎⁎⁎
Informal worker	0.85 (0.81 – 0.89)⁎⁎⁎	0.85 (0.81 – 0.89)⁎⁎⁎	0.85 (0.81 – 0.89)⁎⁎⁎	0.85 (0.81 – 0.89)⁎⁎⁎
Others	0.78 (0.73 – 0.84)⁎⁎⁎	0.78 (0.73 – 0.84)⁎⁎⁎	0.78 (0.73 – 0.84)⁎⁎⁎	0.78 (0.73 – 0.84)⁎⁎⁎
Student	0.83 (0.74 – 0.93)	0.83 (0.74 – 0.93)*	0.83 (0.74 – 0.93)*	0.83 (0.74 – 0.93)*
Quintile (Ref: 1^st^ Quintile)				
2^nd^ Quintile	0.90 (0.86 – 0.95)⁎⁎⁎	0.90 (0.86 – 0.95)⁎⁎⁎	0.91 (0.86 – 0.95)⁎⁎⁎	0.90 (0.86 – 0.95)⁎⁎⁎
3^rd^ Quintile	0.87 (0.82 – 0.91)⁎⁎⁎	0.87 (0.82 – 0.91)⁎⁎⁎	0.87 (0.82 – 0.91)⁎⁎⁎	0.87 (0.82 – 0.91)⁎⁎⁎
4^th^ Quintile	0.78 (0.74 – 0.83)⁎⁎⁎	0.78 (0.74 – 0.83)⁎⁎⁎	0.78 (0.74 – 0.83)⁎⁎⁎	0.78 (0.74 – 0.83)⁎⁎⁎
5^th^ Quintile	0.64 (0.61 – 0.68)⁎⁎⁎	0.64 (0.61 – 0.69)⁎⁎⁎	0.64 (0.61 – 0.69)⁎⁎⁎	0.64 (0.61 – 0.68)⁎⁎⁎
Smoking status (Ref: Everyday)				
Not everyday	1.16 (1.06 – 1.26)⁎⁎	1.16 (1.06 – 1.26)⁎⁎	1.16 (1.06 – 1.26)⁎⁎	1.16 (1.06 – 1.26)⁎⁎
Ex-smoker	1.28 (1.19 – 1.38)⁎⁎⁎	1.28 (1.19 – 1.38)⁎⁎⁎	1.28 (1.19 – 1.38)⁎⁎⁎	1.28 (1.19 – 1.38)⁎⁎⁎
Not smoker	0.73 (0.68 – 0.77)⁎⁎⁎	0.73 (0.68 – 0.77)⁎⁎⁎	0.73 (0.68 – 0.77)⁎⁎⁎	0.73 (0.68 – 0.77)⁎⁎⁎
Alcohol consumption (Ref: Under standard)				
More than standard	1.08 (0.95 – 1.23)	1.08 (0.95 – 1.23)	1.08 (0.95 – 1.23)	1.08 (0.95 – 1.23)
No alcohol	0.66 (0.60 – 0.72)⁎⁎⁎	0.66 (0.60 – 0.72)⁎⁎⁎	0.66 (0.60 – 0.72)⁎⁎⁎	0.66 (0.60 – 0.72)⁎⁎⁎
Physically active	0.92 (0.87 – 0.98)*	0.92 (0.87 – 0.98)*	0.92 (0.87 – 0.98)*	0.92 (0.87 – 0.98)*
Have comorbidities	1.85 (1.81 – 1.89)⁎⁎⁎	1.85 (1.81 – 1.89)⁎⁎⁎	1.85 (1.81 – 1.89)⁎⁎⁎	1.85 (1.81 – 1.89)⁎⁎⁎
Have a family member with psychosis	3.56 (3.19 – 3.97)⁎⁎⁎	3.56 (3.19 – 3.97)⁎⁎⁎	3.56 (3.19 – 3.97)⁎⁎⁎	3.56 (3.19 – 3.97)⁎⁎⁎
Have difficulty accessing healthcare	1.48 (1.41 – 1.55)⁎⁎⁎	1.48 (1.41 – 1.55)⁎⁎⁎	1.48 (1.41 – 1.55)⁎⁎⁎	1.48 (1.41 – 1.55)⁎⁎⁎
Number of observations	572,341	572,341	572,341	572,341

‡ Note: Presented are Odds ratio and 95% Confidence intervals

⁎⁎⁎ p < 0.001

⁎⁎ p < 0.005

⁎ p < 0.05

Age showed a protective effect against mental distress, with those aged 55–74, especially 65–74 (OR: 0.69; p<0.001), having lower odds than those aged 18–24. The most substantial protective effect was observed among those aged 65–74 (OR: 0.69; 95% CI: 0.63–0.76; p < 0.001). Female respondents had nearly twice the odds of reporting mental distress compared to males (OR: 1.98; 95% CI: 1.87–2.10; p < 0.001). Higher levels of education, being married, being employed or retired, and higher household expenditure were independently associated with reduced odds of distress.

Health behaviour variables were also significantly associated with mental distress. Compared to daily smokers, former smokers had higher distress odds than daily smokers, while non-smokers had lower odds. Abstainers from alcohol had lower distress odds than moderate drinkers, while heavy drinkers had higher. Physically active individuals had slightly lower odds of distress.

Health status and psychosocial variables also showed strong effects. Having comorbid chronic illnesses increased the risk of mental distress. Those with a family member diagnosed with psychosis had over three times the odds of mental distress. Difficulty accessing healthcare is also linked to higher mental distress. These findings highlight the complex factors influencing mental distress, including environment, health behaviours, and social vulnerabilities.

## 4 Discussion

This study explored the link between coastal hazards and mental distress among Indonesian adults, and how demographic, socio-economic, health behaviour, and household factors influence these relationships. Individuals in regions affected by climate hazards faced higher odds of mental distress. This aligns with research from multiple countries showing the negative mental health impacts of coastal hazards [27,28]. A regional review across South and Southeast Asia and the Pacific found strong associations between coastal hazard exposure and stress, depression, anxiety, and distress [27], while a study in Bangladesh similarly showed higher psychological distress in high-vulnerability areas [28].

Our analysis showed that people in coastal abrasion areas had experienced slightly higher mental distress, which, despite the small effect size, may have meaningful population impacts or effects from prolonged exposure. This aligns with studies from Bangladesh linking sea-level rise and shoreline abrasion to depression, anxiety, and stress [29]. Similarly, research in eastern Quebec, Canada, found that those exposed to coastal hazards were 2.33 times more likely to report higher stress than unaffected groups [30].

The psychological stress of coastal abrasion exposure may arise from direct trauma and chronic uncertainty. Qualitative evidence from Kerala, India, shows that repeated erosion exposes communities to persistent fear, housing loss, and livelihood insecurity, contributing to hypervigilance, sleep disturbances, and anxiety [31]. The gradual, cumulative nature of abrasion may also produce anticipatory or “pre-traumatic” stress, compounded by social disruption and economic insecurity [29,30].

Another key finding was a higher mental distress among people living in hurricane-exposed areas, consistent with evidence that repeated or severe hurricane exposure has cumulative psychological impacts. Studies from the U.S. show that repeated hurricane exposure is associated with higher levels of depression, anxiety, and post-traumatic stress [32,33]. An extensive systematic review further found that hurricane and flood exposure consistently resulted in greater psychological distress compared with unaffected populations [33]. Long-term follow-up of Hurricane Katrina survivors indicates that pre-disaster trauma may compound these effects over time [34]. Hurricanes can lead to lasting mental distress through direct trauma exposure, including injury, life threat, and bereavement, with more severe or prolonged exposure linked to greater risks of PTSD, anxiety, and depression distress [35,36]. They also disrupt social networks and community cohesion through displacement and loss of support reduces coping capacity and increasing vulnerability to distress [36,37]. Additionally, socioeconomic disruption, such as job loss, financial strain, and unstable living conditions, creates chronic stressors that hinder psychological recovery [35,36].

In contrast to hurricanes and coastal abrasion, tidal flooding was not significantly associated with mental distress in this study, possibly reflecting adaptation or normalisation in repeatedly exposed communities. However, evidence from Indonesia suggests that tidal flooding may be psychologically harmful in a specific context, with high levels of stress and depressive symptoms reported among adolescents and affected households in Semarang and Pekalongan [38,39]. The absence of an association in our analysis may therefore reflect community-level coping and social capital, which have been shown to buffer mental health impacts in both Indonesian [39] and Quebec [30] settings.

Beyond coastal hazards, covariate patterns were consistent with established determinants of mental health. Older age and socio-economic resources were generally protective, while women, chronic conditions, healthcare access difficulties, and household psychosocial stress were associated with higher distress. Health behaviours showed differential associations, with smoking demonstrating stronger and more consistent links to mental distress than alcohol use, suggesting its relevance as a behavioural marker of mental health risk in hazard-exposed populations.

Several limitations should be noted. First, the cross-sectional design prevents causal inference. Second, mental distress was measured with a screening tool rather than a clinical diagnosis, which may under- or overreport symptoms [40]. Third, although some associations were statistically significant, the very large sample size means that small effect sizes are detectable; therefore, results should be interpreted with attention to effect magnitude rather than statistical significance alone. Fourth, the analysis is specific to Indonesia, and findings may not be fully applicable to other coastal areas with different hazards, health systems, or social contexts. Moreover, psychosocial vulnerability was assessed via the Riskesdas indicator of household member psychosis, reflecting available data but not broader mental health stressors. Lastly, as climate risks intensify, our findings may not fully reflect current exposures. Future research should use more recent data and mixed methods to understand the evolving mental health impacts better.

As an exploratory analysis, this study aimed to characterize population-level associations between coastal hazards and mental distress rather than test predefined hypotheses. The purpose was to generate preliminary evidence on an underexamined public health issue and to identify patterns warranting further investigation. The associations observed here therefore provide a foundation for targeted hypotheses in future studies. Our findings suggest that mental distress is a critical outcome of coastal hazards, but it is often overlooked in climate adaptation planning. While global adaptation strategies increasingly emphasise hybrid and community-driven approaches [41,42], Indonesian frameworks such as FOLU Net Sink 2030 remain largely focused on environmental mitigation, with limited attention to mental health impacts [43]. Strengthening community-based and culturally sensitive mental health support, including integration into disaster planning and early detection pathways, could help address these gaps in coastal settings [44,45].

## 5 Conclusion

This study shows that exposure to coastal hazards, particularly abrasion and hurricanes, is linked to greater mental distress among Indonesian adults, even after adjusting for covariates. These findings highlight the importance of integrating mental health into climate adaptation, especially in vulnerable coastal areas. Strengthening community-based services, embedding psychosocial support in disaster planning, and expanding culturally sensitive care are essential to reduce the long-term mental health burden of sea level rise.

## CRediT authorship contribution statement

**Mashita Fajri:** Writing – review & editing, Writing – original draft, Methodology, Investigation, Formal analysis, Data curation, Conceptualization. **Reynalda Fildzah Dessyrianti:** Writing – review & editing, Validation, Investigation. **Sujarwoto Sujarwoto:** Writing – review & editing, Validation, Supervision, Investigation. **Herni Susanti:** Writing – review & editing, Validation, Investigation. **Asri Maharani:** Writing – review & editing, Supervision, Methodology, Investigation, Formal analysis, Conceptualization.

## Declaration of competing interest

The authors declare that they have no known competing financial interests or personal relationships that could have appeared to influence the work reported in this paper.

## Appendix Supplementary materials

Image, application 1

## Footnotes

Supplementary material associated with this article can be found, in the online version, at doi:10.1016/j.joclim.2026.100699.

## References

[1] World Health Organization (2022). https://www.who.int/publications/i/item/9789240045125.

[2] Elneel L., Zitouni M.S, Mukhtar H., Galli P., Al-Ahmad H. (2024). Exploring key aspects of Sea level rise and their implications: an overview. Water.

[3] Jha M.K, Dev M., Azrour M., Mabrouki J., Alabdulatif A., Guezzaz A., Amounas F. (2024). Smart Internet of Things for Environment and Healthcare.

[4] Gaborit P. (2022). Climate adaptation to multi-hazard climate related risks in ten Indonesian cities: ambitions and challenges. Clim Risk Manag.

[5] Nurhidayah L., Davies P., Alam S., Saintilan N., Triyanti A. (2022). Responding to sea level rise: challenges and opportunities to govern coastal adaptation strategies in Indonesia. Marit Stud.

[6] Setiawan H.H, Aldyan R.A, Susantyo B., Irmayani N.R, Habibullah G.M., Almulhim A.I., Leal Filho W., Sharifi A. (2025). Urbanization, Climate Change, and Health: Integrating Strategies for Sustainable and Resilient Cities.

[7] Abu M., Heath S.C, Adger W.N, Codjoe S.NA., Butler C., Quinn T. (2024). Social consequences of planned relocation in response to sea level rise: impacts on anxiety, well-being, and perceived safety. Sci Rep.

[8] Maharani A., Sujarwoto S., Susanti H., Brooks H., Bee P. (2025). Association between climate related hazards and depression among coastal communities in Indonesia. Sci Rep.

[9] Walinski A., Sander J., Gerlinger G., Clemens V., Meyer-Lindenberg A., Heinz A. (2023). The effects of climate change on mental health. Dtsch Arztebl Int.

[10] Charlson F., Ali S., Benmarhnia T., Pearl M., Massazza A., Augustinavicius J. (2021). Climate Change and mental health: a scoping review. Int J Environ Res Public Health.

[11] Clayton S., Crandon T. (2025). Climate change and mental health. Annu Rev Clin Psychol.

[12] Clayton S., Brown L.A. (2024). Climate change and mental health. Jama.

[13] Cosh S.M, Ryan R., Fallander K., Robinson K., Tognela J., Tully P.J (2024). The relationship between climate change and mental health: a systematic review of the association between eco-anxiety, psychological distress, and symptoms of major affective disorders. BMC Psychiatry.

[14] Thompson R., Lawrance E.L, Roberts L.F, Grailey K., Ashrafian H., Maheswaran H. (2023). Ambient temperature and mental health: a systematic review and meta-analysis. Lancet Planet Health.

[15] Klompmaker J.O, Laden F., James P., Benjamin Sabath M., Wu X., Dominici F. (2023). Long-term exposure to summer specific humidity and cardiovascular disease hospitalizations in the US Medicare population. Environ Int.

[16] Sharpe I., Davison C.M. (2021). Climate change, climate-related disasters and mental disorder in low- and middle-income countries: a scoping review. BMJ Open.

[17] Lumban-Gaol J., Sumantyo J.TS., Tambunan E., Situmorang D., Antara I., Sinurat M.E (2024). Sea level rise, land subsidence, and flood disaster vulnerability assessment: a case study in Medan city. Indones Remote Sens.

[18] Christiananda S.A., Santoso E.H. (2024). Assessment of sea level rise and tidal flood vulnerability in the coastal region of Jepara, Central Java, Indonesia. J Mar Resour Coast Manag.

[19] Setiawan H.H, Susantyo B., Irmayani N.R, Habibullah H., Ganti M., Abdullah A., Pal S.C., Chatterjee U., Saha A., Ruidas D. (2025). Climate Change: Conflict and Resilience in the Age of Anthropocene.

[20] Ministry of Health of the Republic of Indonesia (Kemenkes). Basic Health Research [Riset Kesehatan Dasar (Riskesdas)] 2018 [Internet]. Jakarta: Kemenkes; 2007 [updated 2018 Nov 2; cited 2023 Jul 10]. Available from: https://layanandata.kemkes.go.id/katalog-data/riskesdas/ketersediaan-data/riskesdas-2018.

[21] Central Statistics Agency (*Badan Pusat Statistik*). Results of the village potential data collection [Hasil Pendataan Potensi Desa (Podes)] 2018 . Jakarta: *Badan Pusat Statistik* (BPS); 2018 Dec 10. [cited 2025 July 2]. Available from: https://www.bps.go.id/id/pressrelease/2018/12/10/1536/hasil-pendataan-potensi-desa–podes–2018.html.

[22] Idaiani S., Mubasyiroh R., Suryaputri I., Indrawati L., Dharmayanti I. (2022). The validity of the self-reporting questionnaire-20 for symptoms of depression: a sub- analysis of the National Health Survey in Indonesia. Open Access Maced J Med Sci.

[23] Hakim B.A. Identification of abrasion area and prevention system in coastal semarang city. In: The 2nd International Seminar On Sustainable Urban Development 2011; 2011 July 21; Jakarta: ISoSUD 2011; 2011. p. S115-S122. Available from: https://www.repository.trisakti.ac.id/usaktiana/index.php/home/detail/detail_koleksi/5/PRO/penerbit/00000000000000092020#.

[24] Haigh I.D, Nicholls R.J. Coastal flooding. In: Frost M, Baxter J, Buckley P, Dye S, Stoker B, editors. MCCIP Science Review 2017. Plymouth: Marine Climate Change Impacts Partnership; 2017. p. 98-104. doi:10.14465/2017.arc10.009-cof.

[25] World Meteorological Organization (2023). https://wmo.int/content/characteristics-of-tropical-cyclones.

[26] Idaiani S., Fikaris M.F, Chairunnisa G., Fajri M., Anselmi L., Gibson J. (2025). Individual and area-level factors associated with depression in indonesia: a multilevel analysis using the 2018 national basic health research. BMC Public Health.

[27] Kabir S., Newnham E.A, Dewan A., Kok K.QX., Hamamura T. (2024). Climate hazards and psychological health among coastal communities in the Asia-Pacific region: a systematic review of quantitative and qualitative evidence. Health Psychol Rev.

[28] Kabir S., Newnham E.A, Dewan A., Islam M.M, Hamamura T. (2024). Sea-level rise and mental health among coastal communities: a quantitative survey and conditional process analysis. SSM Popul Health.

[29] Kabir S., Newnham E., Dewan A., Islam M.M, Hamamura T. (2024). Psychological health declined during the post-monsoon season in communities impacted by sea-level rise in Bangladesh. Commun Earth Environ.

[30] Boyer-Villemaire U., Kanli C.V, Ledoux G., Gosselin C.-A., Templier S. (2021). Quantifying psychosocial impacts from coastal hazards for cost-benefit analysis in eastern Quebec. Can Front Clim.

[31] Yesodharan R., Jose T.T, Roach E.J. (2021). The lived experience of victims of catastrophic coastal erosion: a cycle of impact, consequence and recovery. Sultan Qaboos Univ Med J.

[32] Garfin D.R, Thompson R.R, Holman E.A, Wong-Parodi G., Silver R.C. (2022). Association between repeated exposure to hurricanes and mental health in a representative sample of Florida residents. JAMA Netw Open.

[33] Miller V.E, Fitch K.V, Swilley-Martinez M.E, Agha E., Alam I.Z, Kavee A.L (2024). Impact of hurricanes and floodings on mental health outcomes within the United States: A systematic review and meta-analysis. Disaster Med Public Health Prep.

[34] Johnson S.T, Mason S.M, Erickson D., Slaughter-Acey J.C, Waters M.C. (2024). Predicting post-disaster post-traumatic stress disorder symptom trajectories: the role of pre-disaster traumatic experiences. Int J Environ Res Public Health.

[35] Heanoy E.Z, Brown N.R. (2024). Impact of natural disasters on mental health: evidence and implications. Healthcare (Basel).

[36] Civelek Y. (2023). The effect of hurricanes on mental health over the long term. Econ Hum Biol.

[37] Kaniasty K. (2020). Social support, interpersonal, and community dynamics following disasters caused by natural hazards. Curr Opin Psychol.

[38] Antika E.R., Sutoyo A., Mulawarman, Munawaroh E., Nadhit G., Pusvitasari Y.N. (2023). Proceedings of the International Seminar on Delivering Transpersonal Guidance and Counselling Services in School (ISDTGCSS 2022).

[39] Widowati I., Harnany A., Amirudin Z. (2020). Psychological well-being Family affected by tidal flood in Pekalongan City. J Educ Health Community Psychol.

[40] Lawrance E., Thompson R., Fontana G., Jennings N. (2021). The impact of climate change on mental health and emotional wellbeing: current evidence and implications for policy and practice. Int Rev Psychiatry.

[41] Lander M.SF., Meguro W., Briones J.I, Castillo I.R, Fletcher C.H. (2024). Envisioning In-situ sea level rise adaptation for coastal cities. Technol| Archit Des.

[42] Bongarts Lebbe T., Rey-Valette H., Chaumillon É, Camus G., Almar R., Cazenave A. (2021). Designing coastal adaptation strategies to tackle sea level rise. Front Mar Sci.

[43] The Ministry of Environment and Forestry of the Republic of Indonesia (2023). https://faolex.fao.org/docs/pdf/ins228605.pdf.

[44] Brooks H., Syarif A.K, Pedley R., Irmansyah I., Prawira B., Lovell K. (2021). Improving mental health literacy among young people aged 11–15 years in Java, Indonesia: the co-development of a culturally-appropriate, user-centred resource (The IMPeTUs Intervention). Child Adolesc Psychiatry Ment Health.

[45] Brooks H., Irmansyah I., Syarif A.K, Pedley R., Renwick L., Rahayu A.P (2023). Evaluating a prototype digital mental health literacy intervention for children and young people aged 11–15 in Java, Indonesia: a mixed methods, multi-site case study evaluation. Child Adolesc Psychiatry Ment Health.

