# Supplementary material for: Mental distress and climate-related coastal hazards: Evidence from national studies in Indonesia
Source: J Clim Chang Health. 2026 May 22;29:100699. doi: 10.1016/j.joclim.2026.100699 (PMC13224060; doi:10.1016/j.joclim.2026.100699)
Supplement: Supplementary file 1 [file mmc1.docx]

# Supplementary Table

**Table S1. Sensitivity analysis: Multivariable logistic regression of the association between residing in coastal hazard areas and mental distress (cut-off ≥6)**

| **Variables** | **Model 1**^‡^ | **Model 2**^‡^ | **Model 3**^‡^ | **Model 4**^‡^ |
| --- | --- | --- | --- | --- |
| Living in coastal hazards | 1.19 (1.04 – 1.36)* |  |  |  |
| Living in district with coastal abrasion |  | 1.02 (1.00 – 1.03)** |  |  |
| Living in district area with hurricanes |  |  | 1.00 (0.99 – 1.00) |  |
| Living in district with tidal flooding |  |  |  | 1.02 (1.00 – 1.03) |
| Age group (Ref: 18-24 years old) |  |  |  |  |
| 25–34 years old | 0.84 (0.81 – 0.88)*** | 0.84 (0.81 – 0.87)*** | 0.84 (0.81 – 0.88)*** | 0.84 (0.81 – 0.88)*** |
| 35–44 years old | 0.87 (0.83 – 0.90)*** | 0.87 (0.83 – 0.90)*** | 0.87 (0.83 – 0.90)*** | 0.87 (0.83 – 0.90)*** |
| 45–54 years old | 0.85 (0.81 – 0.88) *** | 0.85 (0.81 – 0.88)*** | 0.85 (0.81 – 0.88)*** | 0.85 (0.81 – 0.88)*** |
| 55–64 years old | 0.74 (0.71 – 0.77)*** | 0.74 (0.71 – 0.77)*** | 0.74 (0.71 – 0.77)*** | 0.74 (0.71 – 0.77)*** |
| 65–74 years old | 0.75 (0.72 – 0.80)*** | 0.75 (0.72 – 0.80)*** | 0.75 (0.72 – 0.80)*** | 0.75 (0.72 – 0.80)*** |
| 75 years old or above | 0.90 (0.85 – 0.96)* | 0.90 (0.85 – 0.96)** | 0.90 (0.85 – 0.96)** | 0.90 (0.85 – 0.96)* |
| Sex (Ref: male) |  |  |  |  |
| Female | 2.01 (1.95 – 2.08)*** | 2.01 (1.95 – 2.08)*** | 2.01 (1.95 – 2.08)*** | 2.01 (1.95 – 2.08)*** |
| Education (Ref: Primary or lower) |  |  |  |  |
| Junior high school | 0.80 (0.78 – 0.83)*** | 0.80 (0.78 – 0.83)*** | 0.80 (0.78 – 0.83)*** | 0.80 (0.78 – 0.83)*** |
| Senior high school | 0.65 (0.63 – 0.66)*** | 0.65 (0.63 – 0.66)*** | 0.65 (0.63 – 0.66)*** | 0.65 (0.63 – 0.66)** |
| Marital status (Ref: Single) |  |  |  |  |
| Married | 0.78 (0.75 – 0.81)*** | 0.78 (0.75 – 0.81)*** | 0.78 (0.75 – 0.81)*** | 0.78 (0.75 – 0.81)*** |
| Divorced or widowed | 1.07 (1.02 – 1.12)** | 1.07 (1.02 – 1.12)** | 1.07 (1.02 – 1.12)** | 1.07 (1.02 – 1.12)** |
| Occupation (Ref: Jobless) |  |  |  |  |
| Employed or retired | 0.66 (0.64 – 0.69)*** | 0.66 (0.64 – 0.69)*** | 0.66 (0.64 – 0.69)*** | 0.66 (0.64 – 0.69)*** |
| Self-employed | 0.82 (0.79 – 0.84)*** | 0.82 (0.79 – 0.84)*** | 0.82 (0.79 – 0.84)*** | 0.82 (0.79 – 0.84)*** |
| Informal worker | 0.90 (0.88 – 0.93)*** | 0.90 (0.88 – 0.93)*** | 0.90 (0.88 – 0.93)*** | 0.85 (0.81 – 0.89)*** |
| Others | 0.80 (0.77 – 0.83)*** | 0.80 (0.77 – 0.83)*** | 0.80 (0.77 – 0.83)*** | 0.90 (0.88 – 0.93)*** |
| Student | 0.94 (0.88 – 1.00) | 0.94 (0.88 – 1.00) | 0.94 (0.88 – 1.00) | 0.94 (0.88 – 1.00) |
| Quintile (Ref: 1^st^ Quintile) |  |  |  |  |
| 2^nd^ Quintile | 0.98 (0.95 – 1.01) | 0.98 (0.95 – 1.01) | 0.98 (0.95 – 1.01) | 0.98 (0.95 – 1.01) |
| 3^rd^ Quintile | 0.92 (0.89 – 0.95)*** | 0.92 (0.89 – 0.95)*** | 0.92 (0.89 – 0.95)*** | 0.92 (0.89 – 0.95)*** |
| 4^th^ Quintile | 0.87 (0.84 – 0.89)*** | 0.87 (0.84 – 0.89)*** | 0.87 (0.84 – 0.89)*** | 0.87 (0.84 – 0.89)*** |
| 5^th^ Quintile | 0.73 (0.71 – 0.76)*** | 0.73 (0.71 – 0.76)*** | 0.73 (0.71 – 0.76)*** | 0.73 (0.71 – 0.76)*** |
| Smoking status (Ref: Everyday) |  |  |  |  |
| Not everyday | 1.04 (0.99 – 1.09) | 1.04 (0.99 – 1.09) | 1.04 (0.99 – 1.09) | 1.04 (0.99 – 1.09) |
| Ex-smoker | 1.18 (1.13 – 1.23)*** | 1.18 (1.13 – 1.23)*** | 1.18 (1.13 – 1.23)*** | 1.18 (1.13 – 1.23)*** |
| Not smoker | 0.71 (0.69 – 0.74)*** | 0.71 (0.69 – 0.74)*** | 0.71 (0.69 – 0.74)*** | 0.71 (0.69 – 0.74)*** |
| Alcohol consumption (Ref: Under standard) |  |  |  |  |
| More than standard | 1.02 (0.95 – 1.11) | 1.02 (0.95 – 1.11) | 1.03 (0.95 – 1.11) | 1.02 (0.95 – 1.11) |
| No alcohol | 0.66 (0.63 – 0.70)*** | 0.66 (0.63 – 0.70)*** | 0.66 (0.63 – 0.70)*** | 0.66 (0.63 – 0.70)*** |
| Physically active | 0.91 (0.88 – 0.94)*** | 0.91 (0.88 – 0.94)*** | 0.91 (0.88 – 0.94)*** | 0.91 (0.88 – 0.94)*** |
| Have comorbidities | 1.78 (1.75 – 1.80)*** | 1.78 (1.75 – 1.80)*** | 1.78 (1.75 – 1.80)*** | 1.78 (1.75 – 1.80)*** |
| Have a family member with psychosis | 2.53 (2.34 – 2.74)*** | 2.53 (2.34 – 2.74)*** | 2.53 (2.34 – 2.74)*** | 2.53 (2.34 – 2.74)*** |
| Have difficulty accessing healthcare | 1.39 (1.35 – 1.43)*** | 1.39 (1.35 – 1.43)*** | 1.39 (1.35 – 1.43)*** | 1.39 (1.35 – 1.43)*** |
| Number of observations | 572,341 | 572,341 | 572,341 | 572,341 |

Note: ^‡^Presented are Odds ratio and 95% Confidence intervals; ***p < 0.001, **p < 0.005, * p < 0.05
